# 热脱附-气流调制全二维气相色谱-飞行时间质谱测定环境大气中中等挥发性有机物

**DOI:** 10.3724/SP.J.1123.2025.07009

**Published:** 2026-04-08

**Authors:** Rui WANG, Yingjie LI, Jiahua WANG, Changwen MA, Jiakui JIANG

**Affiliations:** 1.生态环境部城市大气复合污染成因与防治重点实验室，上海市环境科学研究院，上海 200233; 1. Key Laboratory of Formation and Prevention of Urban Air Pollution Complex，Ministry of Ecology and Environment，Shanghai Academy of Environmental Sciences，Shanghai 200233，China; 2.上海第二工业大学，上海 201209; 2. Shanghai Polytechnic University，Shanghai 201209，China; 3.思聚仪器仪表（上海）有限公司，上海 200233; 3. Schauenburg Analytics Solutions （Shanghai） Co. ，Ltd. ，Shanghai 200233，China

**Keywords:** 热脱附, 气流调制, 全二维气相色谱, 飞行时间质谱, 中等挥发性有机物, 环境大气, thermal desorption, flow modulation, comprehensive two-dimensional gas chromatography （GC×GC）, time-of-flight mass spectrometry （TOF-MS）, intermediate volatility organic compounds （IVOCs）, ambient atmosphere

## Abstract

环境大气中中等挥发性有机物（IVOCs）组成复杂；因受传统一维气相色谱柱分离能力限制，大部分IVOCs以未分离的色谱峰从色谱柱流出洗脱，因此无法将IVOCs准确识别和定量，制约了对其准确溯源。针对以上问题，本研究基于热脱附-气流调制全二维气相色谱-飞行时间质谱（TD-FM GC×GC-TOF MS）技术，通过优化气流调制周期的填充时间和吹扫时间，建立了一种高效分离和定量环境大气中IVOCs的分析方法。以ZB-5HT（20 m×0.18 mm×0.18 μm）为一维色谱柱、BP-50（5 m×0.25 mm×0.20 μm）为二维色谱柱分离IVOCs，设置填充时间2 870 ms、吹扫时间130 ms。在优化的分析条件下，69个定量目标物在1~20 ng/管范围内呈现良好的线性关系，相关系数为0.922 3~0.998 4等；方法检出限为0.010 7~0.410 1 ng/m^3^；目标物在1 ng/管加标水平下的回收率为80.4%~136.0%，在1、3和10 ng/管的相对标准偏差分别为4.5%~33.9%、3.2%~19.9%、3.5%~18.6%。利用该方法测定上海城区环境大气中IVOCs的质量浓度和组成分布，结果显示，总IVOCs质量浓度在8.6~61.1 μg/m^3^，样品中检测到可识别有机物853个，对总IVOCs质量浓度贡献可高达96.2%，且新识别的芳香烃、Cl-IVOCs、O-IVOCs等有机物与常规监测目标物表现出不同排放来源特征。因此，该方法不仅为测定环境大气复杂IVOCs提供了一种可靠的技术手段，也为大气中IVOCs的精准溯源提供了数据支撑，还可应用于其他环境介质中IVOCs的测定，为多介质中IVOCs污染特征研究提供有力的技术支持。

## Abstract (en)

Atmospheric intermediate volatile organic compounds （IVOCs） are complex mixtures. Due to the limited separation and identification capabilities of one-dimensional gas chromatography （^1^D-GC）， a large portion of atmospheric IVOCs—which often elute as unresolved or complex peaks—are categorized as an unspeciated complex mixture （UCM）. This constrains the accurate apportionment of atmospheric IVOCs. To address this problem， an alternative analytical method has been developed utilizing thermal desorption-flow modulation comprehensive two-dimensional gas chromatography coupled with time-of-flight mass spectrometry （TD-FM GC×GC-TOF MS） to measure atmospheric IVOCs. Analytical optimization focused on fill and flush time within a 3-second modulation period， with optimal values determined as 2 870 ms and 130 ms， respectively， to maximize separation efficiency and signal response. Compounds were separated on a ZB-5HT column （20 m×0.18 mm×0.18 μm） as the first dimension， with a BP-50 column （5 m×0.25 mm×0.20 μm） serving as the second dimension. Helium was employed as the carrier gas with column pressures maintained at 254.4 kPa （first dimension） and 202.1 kPa （second dimension）. The following temperature program was used： 50 ℃ for 10 min， then increased to 300 ℃ at 10 ℃/min， with the final temperature held for 20 min. The MS was operated in electron impact （EI， 70 eV） ionization mode. Both the ion source and transfer line temperatures were set at 300 ℃. MS signals were recorded in full scan mode with a scan range of *m/z* 40 to *m/z* 800. During thermal desorption， the desorption temperature was set at 320 ℃， and the cold trap temperature was maintained at -10 ℃ to capture desorbed targets. The secondary desorption flow rate was set at 16 mL/min. Under these optimized conditions， the method demonstrated acceptable linearity， with correlation coefficients ranging from 0.922 3 to 0.998 4， for 69 selected targets spanning 1 to 20 ng/tube， with method detection limits （spiked at 1 ng/tube） between 0.010 7 and 0.410 1 ng/m^3^， with recoveries ranging from 80.4% to 136.0%. Relative standard deviation values among replicate samples （*n*=7） spiked at 1 ng/tube， 3 ng/tube， and 10 ng/tube levels were around 4.5%-33.9%， 3.2%-19.9% and 3.5%-18.6%， respectively. Application of the method to atmospheric IVOC samples collected from an urban site of Shanghai， revealed total IVOCs mass concentrations of 8.6-61.1 μg/m^3^. Of these， mass concentrations of 853 individual species identified by the present method comprised 96.2% of total IVOCs. Consequently，the newly developed method improved the identification rate for UCM. Importantly， the newly speciated aromatics， chlorinated IVOCs， and oxygenated IVOCs exhibited different emission sources distinct from those routinely monitored compounds. Therefore， this study established a reliable method for determining atmospheric IVOCs， offering strong data support for precise source apportionment of complex IVOCs in the atmosphere. Additionally， this method can be used to monitor IVOCs across various environmental metrics， thereby providing essential data to support the management and control of these compounds.

中等挥发性有机物（IVOCs）是指有效饱和质量浓度为10^3^~10^6^ μg/m^3^的一类化合物^［1］^。排放进入环境大气后，IVOCs可被高效地氧化生成二次有机气溶胶（SOA）^［2］^，被视为PM_2.5_的重要前体物。因此，准确识别环境大气中IVOCs的污染特征，可为制定相关管控措施提供基础数据支撑。然而，环境大气中IVOCs的组分非常复杂，尤其随着碳原子数的增加，IVOCs种类呈指数增长态势^［3］^。通常，IVOCs采用一维气相色谱-质谱（GC-MS）方法进行测定，因受单个色谱柱分离能力的限制，IVOCs组分很难被完全分离^［4，5］^。目前，仅有一小部分IVOCs，如烷烃（alkanes）、多环芳烃（PAHs）、邻苯二甲酸酯类（PAEs）等^［6］^，被GC-MS识别和定量，超过70%的组分以共洗脱方式从色谱柱流出^［7，8］^，研究者把这部分称为未识别组分（UCM）^［7-9］^。UCM一般采用正构烷烃的响应因子与UCM的总离子流色谱峰面积（TIC）比值进行半定量估算^［7，8］^，但因为不能识别具体组分，难以对UCM精准溯源^［8］^。全二维气相色谱技术（GC×GC）通过调制器连接两个极性不同的色谱柱，经一维色谱柱分离的组分在二维色谱柱上进一步分离，提高了复杂混合物的分离效果^［10］^。调制器作为连接两个色谱柱的核心部件，具有对一维色谱柱分离的目标物进行捕获、聚集和释放进入二维色谱柱的功能^［11］^。根据调制原理不同，调制器分为热调制和气流调制两种模式^［12］^。热调制通过低温聚集和高温解吸实现目标物的捕集和释放^［13］^。根据低温聚集温度不同，可实现>C_3_（-40~20 ℃）^［14］^和>C_7_（-20~10 ℃）^［12］^有机物的分析。与热调制技术不同，气流调制技术利用周期性的气流将一维色谱柱流出的目标物吹扫进入二维色谱柱，实现目标物在调制器内捕集和释放^［13］^。Duhamel等^［15］^比较了气流调制与热调制技术对稠油样品中有机物组分的识别效果，发现气流调制器对>C_14 _的组分有着更窄的峰宽与更高的峰值容量。

随着二维分离技术优势凸显，全二维气相色谱-质谱（GC×GC-MS）技术已广泛应用于测定污水^［16-18］^、土壤^［19，20］^、空气^［21-23］^等环境介质的有机物。对不同介质中的目标物，主要采用溶剂萃取法进行浓缩预处理，再使用GC×GC-MS进行分析^［16，24，25］^。然而，溶剂萃取法操作步骤烦琐、费时费力，且易引入人为误差、造成样品潜在污染^［26］^。不同于溶剂萃取，热脱附（TD）是一种气体萃取技术，即在一定的气流下加热吸附剂解吸目标物，具有操作简单、省时省力、不易引入杂质干扰等优点^［27］^。目前，已有少数研究利用TD联合GC×GC-MS（TD-GC×GC-MS）分析了家庭烹饪和重型柴油车排放的IVOCs以及环境大气中IVOCs的组成特征^［28-30］^，将可识别IVOCs组分相对于一维GC技术（5%~20%）提高至85%以上^［7，8］^。但是，上述研究主要以使用热调制全二维GC技术为主，新兴的TD-气流调制GC×GC-MS相关研究鲜有报道。本研究在优化气流调制器运行参数的基础上，建立了基于TD-气流调制GC×GC-MS测定环境大气中IVOCs的方法，并将建立的方法用于测定上海城区环境大气中IVOCs的组成分布情况。

## 1 实验部分

### 1.1 仪器、试剂与材料

ATD650热脱附仪（美国PerkinElmer公司），Trace 1310气相色谱仪（美国Thermo Fisher公司），全二维气流调制器（Insight）（英国SepSolve Analytical公司），BenchTOF Evolve飞行时间质谱仪、空不锈钢TD采样管、便携式采样泵（Acti-VOC）和TC-20^TM^不锈钢热脱附管老化仪（英国Markes公司），质量流量计（Sensidyne）（美国Gilian公司）。

69个IVOCs标准样品购自Sigma-Aldrich公司（上海）与AccuStandard公司，6个同位素内标溶液包括正十二烷-d_26_（C_12_-d_26_）、正十五烷-d_32_（C_15_-d_32_）、正十九烷-d_40_（C_19_-d_40_）、萘-d_8_（Nap-d_8_）、苊-d_10_（Ace-d_10_）、菲-d_10_（Phe-d_10_）以及己烷溶剂（HPLC级）购自Sigma-Aldrich公司（上海），具体见表1。

**表1:** IVOCs标准样品规格及来源

Standard sample	Solvent	Mass concentration	Supplier
*n*-Alkanes SM	dichloromethane	500 μg/mL	Sigma-Aldrich
*i*-Alkanes Std.	dichloromethane	2000 μg/mL	Sigma-Aldrich
Alkyl cycloalkanes Std.	isooctane	1000 μg/mL	AccuStandard
Alkenes Std.	dichloromethane	1000 μg/mL	Sigma-Aldrich
PAHs SM	benzene-dichloromethane （1∶1）	2000 μg/mL	Sigma-Aldrich
Alkyl naphthalenes Std.	benzene-dichloromethane （1∶1）	1000 μg/mL	Sigma-Aldrich
Tetralines Std.	benzene-dichloromethane （1∶1）	1000 μg/mL	Sigma-Aldrich
Nitro-PAHs Std.	chloroform	100 μg/mL	Sigma-Aldrich
SRAs Std.	chloroform	2000 μg/mL	Sigma-Aldrich
Cl-IVOCs SM	dichloromethane	2000 μg/mL	AccuStandard
O-IVOCs Std.	dichloromethane	2000 μg/mL	Sigma-Aldrich
PAHs IS	acetonitrile	2000 μg/mL	Sigma-Aldrich
C_12_-d_26_	/	0.864 g/mL	Sigma-Aldrich
C_15_-d_32_	/	0.883 g/mL	Sigma-Aldrich
C_19_-d_40_	/	0.904 g/mL	Sigma-Aldrich
Hexane	/	HPLC， purity≥99.7%	Sigma-Aldrich

*n*-Alkanes SM： *n*-alkanes standard mixture； SM： standard mixture； *i*-Alkanes： iso-alkanes； Std.： standard； PAHs SM： polycyclic aromatic hydrocarbons standard mixture； SRAs： single ring aromatics； Cl-IVOCs SM： chlorinated intermediate volatile organic compounds standard mixture； O-IVOCs： oxygenated intermediate volatile organic compounds； PAHs IS： internal standard of polycyclic aromatic hydrocarbons； C_12_-d_26_： dodecane-d_26_； C_15_-d_32_： pentadecane-d_32_； C_19_-d_40_： nonadecane-d_40_； HPLC： high performance liquid chromatography grade.

吸附剂Glassbeads（212~300 μm）、Carbopack C（60/80目）、CarbopackB（60/80目）和TD管内金属网、玻璃纤维购自Sigma-Aldrich公司（上海）；高纯氮气（99.999%）和高纯氦气（99.999%）购自林德工业气体有限公司（上海）。

### 1.2 实验方法

#### 1.2.1 标准溶液的配制

分别取一定量的不同标准溶液混合于己烷溶剂中，经己烷逐级稀释，得到质量浓度分别为1、3、5、10、15和20 mg/L的系列混合标准溶液；将6个内标溶液混合于己烷溶剂，经己烷逐级稀释后，得到质量浓度为4.5 mg/L的内标混合溶液。

#### 1.2.2 吸附管的老化与保存

TD管内吸附剂在实验室内装填完成，详细信息可查阅文献［^31^］。新填装完成的TD管需使用老化仪老化，在350 ℃下以100 mL/min的高纯氦气吹扫5 h（初次使用）或1 h（再次使用）。老化后的TD管两端分别用黄铜盖密封，以铝箔纸包覆，于4 ℃冰箱保存备用。

#### 1.2.3 实验室样品准备

先在TD管中加入1.0 μL系列混合标准溶液，再加入1.0 μL 4.5 mg/L的内标混合溶液，以50 mL/min的流速用高纯氮气吹扫5 min，除去溶剂。

#### 1.2.4 样品采集

在上海城区上海市环境科学研究院楼顶采集环境大气样品。该采样点距离地面约30 m。采样时间为2022年10月10日~16日，每日分4个采样时段，分别为6：30~9：00、9：00~16：30、16：30~19：00和19：00~次日6：30。采样时，IVOCs（气态+颗粒态）由TD管连接一个便携式采样泵进行采集。在采样开始前和结束后，使用质量流量计对采样泵的实际采样流速进行校准。

### 1.3 分析条件

#### 1.3.1 气流调制条件

基于气流调制技术的全二维气相色谱调制器主要由不锈钢材质的七通接口连通而成。各接口分别连接切换阀（switching valve）、一维色谱柱（^1^D column）、样品环（sample loop）、二维色谱柱（^2^D column）、放空柱（bleed line）。

如图1所示，经一维色谱柱分离的目标物由高纯氦气运载填充至样品环中，填充一定时间后，利用高纯氦气对填充在样品环中的目标物进行反向吹扫，以确保样品环内目标物全部由高纯氦气载入二维色谱柱。填充时间与吹扫时间的加和为气流调制的一个调制周期，调制周期默认设置为3 s。放空柱用于保持气流调制系统的压力平衡^［32］^。本研究中设置气流调制填充时间为2 870 ms，吹扫时间为130 ms。

**图1:**
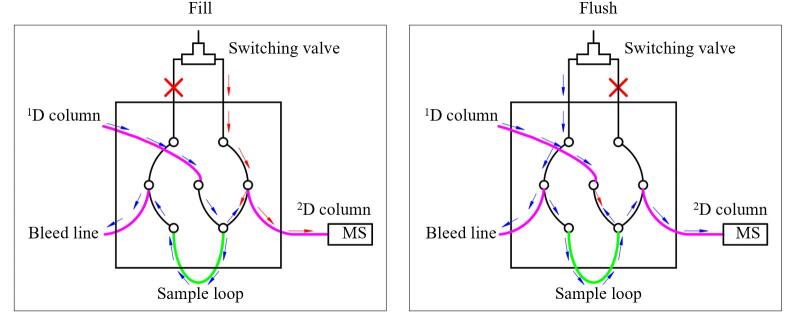
气流调制器工作示意图

#### 1.3.2 热脱附条件

在热脱附前，使用高纯氦气以60 mL/min的流速在室温下吹扫TD样品管1 min，以去除样品中的水分和氧气。随后，以高纯氦气为载气，在320 ℃下以60 mL/min的流速进行首次热脱附（20 min），释放的目标物在冷阱管（-10 ℃）内富集。热脱附完成后，冷阱管快速升至320 ℃，在16 mL/min的流速下进行二次热脱附（10 min）。二次热脱附的目标物经由传输管线传输至气相色谱柱前端，传输线温度设置为300 ℃。

#### 1.3.3 气相色谱-质谱条件

色谱条件 一维色谱柱ZB-5HT（20 m×0.18 mm×0.18 μm，美国Phenomenex公司）；二维色谱柱BP-50（5 m×0.25 mm×0.20 μm，中国NanoChrom公司）。载气：高纯氦气；恒压模式：一维色谱柱压力254.4 kPa，二维色谱柱压力202.1 kPa；升温程序：初始温度50 ℃，保持10 min，然后以10 ℃/min升至300 ℃，保持20 min。

质谱条件 电子轰击源（EI）；电离电压：70 eV；离子源温度：300 ℃；传输线温度：300 ℃。全扫描模式：*m/z* 40~800。

### 1.4 定量计算

采用内标法进行定量计算，各化合物的内标信息见表2。

**表2:** 定量可识别IVOCs的名称、缩写、定量离子、保留时间和参考用内标物

No.	Compound	Abbreviation	QI （*m/z*）	RT Ⅰ/s	RT Ⅱ/s	IS
	**Alkanes**					
	*n*-Alkanes					
1	*n*-dodecane	C_12_	57	1008.6	1.14	C_12_-d_26_
2	*n*-tridecane	C_13_	57	1113.0	1.07	C_12_-d_26_
3	*n*-tetradecane	C_14_	57	1203.0	1.13	C_15_-d_32_
4	*n*-pentadecane	C_15_	57	1284.0	1.20	C_15_-d_32_
5	*n*-hexadecane	C_16_	57	1360.2	1.30	C_15_-d_32_
6	*n*-heptadecane	C_17_	57	1431.6	1.44	C_19_-d_40_
7	*n*-octadecane	C_18_	57	1503.6	1.55	C_19_-d_40_
8	*n*-nonadecane	C_19_	57	1570.2	1.68	C_19_-d_40_
9	*n*-eicosane	C_20_	57	1630.2	1.79	C_19_-d_40_
10	*n*-heneicosane	C_21_	57	1689.6	1.93	C_19_-d_40_
11	*n*-docosane	C_22_	57	1747.2	2.02	C_19_-d_40_
	*i*-Alkanes					
12	pristane		57	1437.0	1.24	C_19_-d_40_
13	phytane		57	1506.6	1.40	C_19_-d_40_
	Alkyl cycloalkanes					
14	hexylcyclohexane	C_6_CH	83	1047.6	1.35	C_12_-d_26_
15	octylcyclohexane	C_6_CO	83	1242.0	1.48	C_15_-d_32_
16	nonylcyclohexane	C_6_CN	83	1327.2	1.62	C_15_-d_32_
	**Alkenes**					
17	1-dodecene		55	996.0	1.11	C_12_-d_26_
18	1-tridecene		55	1101.6	1.11	C_12_-d_26_
19	1-cetene		55	1430.4	1.41	C_19_-d_40_
20	1-octadecene		55	1497.6	1.67	C_19_-d_40_
	**Aromatics**					
	PAHs					
21	naphthalene	Nap	128	990.0	0.94	Nap-d_8_
22	acenaphthylene	Acp	152	1253.4	1.30	Ace-d_10_
23	acenaphthene	Ace	153	1282.8	1.14	Ace-d_10_
24	fluorene	Flu	166	1362.0	1.32	Ace-d_10_
25	phenanthrene	Phe	178	1506.0	2.34	Phe-d_10_
26	anthracene	Ant	178	1512.0	2.18	Phe-d_10_
27	fluoranthene	Fluo	202	1686.0	0.17	Phe-d_10_
28	pyrene	Pyr	202	1717.2	0.64	Phe-d_10_
	Alkyl naphthalenes					
29	2-methylnaphthalene	2-MN	142	1113.0	0.53	Nap-d_8_
30	1-methylnaphthalene	1-MN	142	1130.4	0.59	Nap-d_8_
31	1，3-dimethylnaphthalene	1，3-DMN	156	1225.2	0.58	Ace-d_10_
32	1，6-dimethylnaphthalene	1，6-DMN	156	1225.2	0.57	Ace-d_10_
33	1，4-dimethylnaphthalene	1，4-DMN	156	1242.0	0.63	Ace-d_10_
34	2，3-dimethylnaphthalene	2，3-DMN	141	1254.0	0.77	Ace-d_10_
35	1，2-dimethylnaphthalene	1，2-DMN	156	1241.4	0.63	Ace-d_10_
	nitro-PAHs					
36	1-nitronaphthalene	1-N-Nap	127	1380.0	2.13	Ace-d_10_
37	2-nitronaphthalene	2-N-Nap	127	1410.0	2.35	Phe-d_10_
	Tetralines					
38	1-methyltetralin	1-MT	131	1035.6	2.91	Nap-d_8_
39	1，4-methylteralin	1，4-DMT	145	1098.0	2.66	Nap-d_8_
40	2，7-methylteralin	2，7-DMT	118	1098.6	2.66	Nap-d_8_
41	2，6-methylteralin	2，6-DMT	118	1131.0	2.71	Nap-d_8_
42	1，1，6-methylteralin	1，1，6-TMT	159	1166.4	2.58	Ace-d_10_
43	1，5，8-methylteralin	1，5，8-TMT	159	1269.0	2.96	Ace-d_10_
44	2，5，8-methylteralin	2，5，8-TMT	159	1269.0	2.95	Ace-d_10_
45	2，2，5，7-methylteralin	2，2，5，7-TMT	132	1257.0	2.60	Ace-d_10_
	SRAs					
46	octylbenzene	C_8_-AB	91	1268.4	2.09	Ace-d_10_
47	decylbenzene	C_10_-AB	92	1422.6	2.37	Phe-d_10_
48	undecylbenzene	C_11_-AB	92	1497.0	2.43	Phe-d_10_
49	dodecylbenzene	C_12_-AB	92	1563.0	2.59	Phe-d_10_
50	tridecylbenzene	C_13_-AB	92	1629.0	2.67	Phe-d_10_
51	tetradecylbenzene	C_14_-AB	92	1689.6	2.81	Phe-d_10_
52	benzothiazole	BTH	134	1061.4	1.03	Nap-d_8_
53	biphenyl		154	1194.6	0.66	Ace-d_10_
54	1-phenylnaphthalene	1-phenylNAP	204	1557.6	1.98	Phe-d_10_
	**Cl-IVOCs**					
55	hexachlorobutadiene	HCBD	225	1026.6	2.12	C_12_-d_26_
56	1，2，4-trichlorobenzene	1，2，4-triCB	180	984.6	0.26	C_12_-d_26_
57	1，2，3-trichlorobenzene	1，2，3-triCB	180	1024.2	0.31	C_12_-d_26_
58	2，4，5-trichlorotoluene	2，4，5-triCB	159	1109.4	2.99	C_12_-d_26_
59	1，2，3，4-tetrachlorobenzene	1，2，3，4-tetraCB	216	1141.8	0.14	C_12_-d_26_
60	1，2，3，5-tetrachlorobenzene	1，2，3，5-tetraCB	216	1141.8	0.14	C_12_-d_26_
61	1，2，4，5-tetrachlorobenzene	1，2，4，5-tetraCB	216	1189.8	0.41	C_15_-d_32_
62	pentachlorobenzene	PCB	284	1308.0	0.57	C_15_-d_32_
63	hexachlorobenzene	HCB	250	1452.0	1.07	C_19_-d_40_
64	octachlorostyrene	OCS	308	1668.6	1.03	C_19_-d_40_
	**O-IVOCs**					
65	dibenzofuran	DBF	168	1308.6	1.18	C_15_-d_32_
66	diphenyl sulfone	DPS	125	1605.0	0.34	C_19_-d_40_
67	xanthone	XT	196	1563.0	2.81	C_19_-d_40_
68	benzophenone	BP	105	1398.0	1.63	C_15_-d_32_
69	2‚6-diphenylphenol	2，6-DPP	246	1563.0	2.60	C_19_-d_40_

RT Ⅰ： retention time of the 1^st^ dimensional chromatography； RT Ⅱ： retention time of the 2^nd^ dimensional chromatography.

将总IVOCs分为可识别组分和UCM两部分，可识别组分进一步分为定量和半定量两类目标物。定量可识别组分（表2）指采用商用标准样品制备的校正曲线进行定量的化合物；半定量可识别组分（表3）为采用与其保留时间相近且结构类似目标物的校正曲线进行定量的化合物^［29］^。因受到标准样品可获取性限制，半定量可识别组分数据质控指标参考相应的定量目标物。

**表3:** IVOCs半定量估算的参考物种

Bin	Group	Semiquantitative compound	Bin	Group	Semiquantitative compound	Bin	Group	Semiquantitative compound
B_12_	alkanes	C_12_	B_16_	alkanes	C_16_	B_20_	alkanes	C_20_
	alkenes	C_12_		alkenes	C_16_		alkenes	C_20_
	aromatics	Nap		aromatics	Flu		aromatics	C_12_-AB
	Cl-IVOCs	HCBD		Cl-IVOCs	PCB		Cl-IVOCs	OCS
	O-IVOCs	C_12_		O-IVOCs	C_16_		O-IVOCs	C_20_
B_13_	alkanes	C_13_	B_17_	alkanes	C_17_	B_21_	alkanes	C_21_
	alkenes	C_13_		alkenes	C_17_		alkenes	C_21_
	aromatics	2-MN		aromatics	C_10_-AB		aromatics	Fluo
	Cl-IVOCs	2，4，5-triCB		Cl-IVOCs	HCB		Cl-IVOCs	OCS
	O-IVOCs	C_13_		O-IVOCs	C_17_		O-IVOCs	C_21_
B_14_	alkanes	C_14_	B_18_	alkanes	C_18_	B_22_	alkanes	C_22_
	alkenes	C_14_		alkenes	C_18_		alkenes	C_22_
	aromatics	1‚3-DMNap		aromatics	Phe		aromatics	Fluo
	Cl-IVOCs	1，2，3，4-tetraCB		Cl-IVOCs	HCB		Cl-IVOCs	OCS
	O-IVOCs	C_14_		O-IVOCs	C_18_		O-IVOCs	C_22_
B_15_	alkanes	C_15_	B_19_	alkanes	C_19_			
	alkenes	C_15_		alkenes	C_19_			
	aromatics	Ace		aromatics	C_12_-AB			
	Cl-IVOCs	PCB		Cl-IVOCs	OCS			
	O-IVOCs	C_15_		O-IVOCs	C_19_			

UCM的定量参考文献［^29^，^33^］：将样品的TIC图根据正构烷烃的保留时间分成*n*个区间（B *_n_* ），*n*与分布在该区间内的正构烷烃碳原子数相同。半定量可识别组分采用分布在同一区间内的同系物响应因子进行定量，UCM则采用分布在同一区间内正构烷烃的响应因子进行定量。具体如下：


$$t_{n,binstart}=t_{n}-\frac{t_{n}-t_{n-1}}{2}；t_{n,binend}=t_{n}+\frac{t_{n+1}-t_{n}}{2}$$ （1）

$$M_{semi-quantified}=\sum_{i=1}^{n}\sum_{j=1}^{m}\frac{TA_{TIC,m,B\left(j\right)}}{RF_{TIC,j-homolog}}$$ （2）

$$M_{UCM}=\sum_{i}^{n}\frac{TA_{TIC,UCM,B(n)}}{RF_{TIC,n-alkane}}$$ （3）

$$M_{total}=M_{quantified}+M_{semi-quantified}+M_{UCM}$$ （4）

式中：*M*_total_、*M*_quantified_、*M*_semi-quantified_和*M*_UCM_分别为IVOCs的总质量、定量可识别IVOCs质量、半定量可识别IVOCs质量和UCM质量；*t_n_*_，binstart_和*t_n_*_，binend_分别表示区间B *_n_* 的起始和结束时间；*t_n_* 、*t_n_*_-1_和*t_n_*_+1_分别为含有*n*、*n*-1和*n*+1碳原子正构烷烃的保留时间；TA_TIC，_ *_m_*_，B（_ *_j_*_）_和TA_TIC，UCM，B（_ *_n_*_）_分别为B *_n_* 区间可识别半定量目标物和UCM的TIC峰面积；RF_TIC，_ *_j_*_-homolog_为B *_n_* 区间用于半定量目标物*j*组分的响应因子；RF_TIC，_ *_n_*_-alkane_为B *_n_* 区间用于半定量UCM的正构烷烃的响应因子。

为去除样品在准备、采集和储存过程中可能引入的污染误差，采样期间，将空白TD管带至采样现场，连接到采样泵上但不进行采样，随后立即将TD管拆卸，带回实验室分析。本研究中所有目标物质量浓度均扣除了背景值。

## 2 结果与讨论

### 2.1 气流调制器运行参数优化

气流调制周期为填充时间和吹扫时间的总和，是气流调制的关键。在固定的调制周期内，填充时间和吹扫时间的设定值会影响目标物的分离和响应效果^［34］^。因此，本研究比较了不同时间参数组合（填充时间/吹扫时间：2 850 ms/150 ms、2 870 ms/130 ms、2 885 ms/115 ms、2 900 ms/100 ms、2 920 ms/80 ms）对11个正构烷烃（C_12_~C_22_）、2个支链烷烃（姥鲛烷（pristane）和植烷（phytane））、3个烷基环烷烃（C_6_CH、C_6_CO和C_6_CN）等代表性化合物在5 ng水平下的色谱图效果和色谱峰面积响应的影响。结果如图2所示，目标物峰的拖尾现象随着吹扫时间的减少得到明显改善。然而，过短的吹扫时间（<130 ms）会导致峰面积明显降低。值得注意的是，当吹扫时间为130 ms（对应填充时间2 870 ms）时，目标化合物的峰形改善效果达到最佳，同时色谱峰响应最强。因此，本研究在后续分析中，均采用填充时间2 870 ms与吹扫时间130 ms的参数组合。

**图2:**
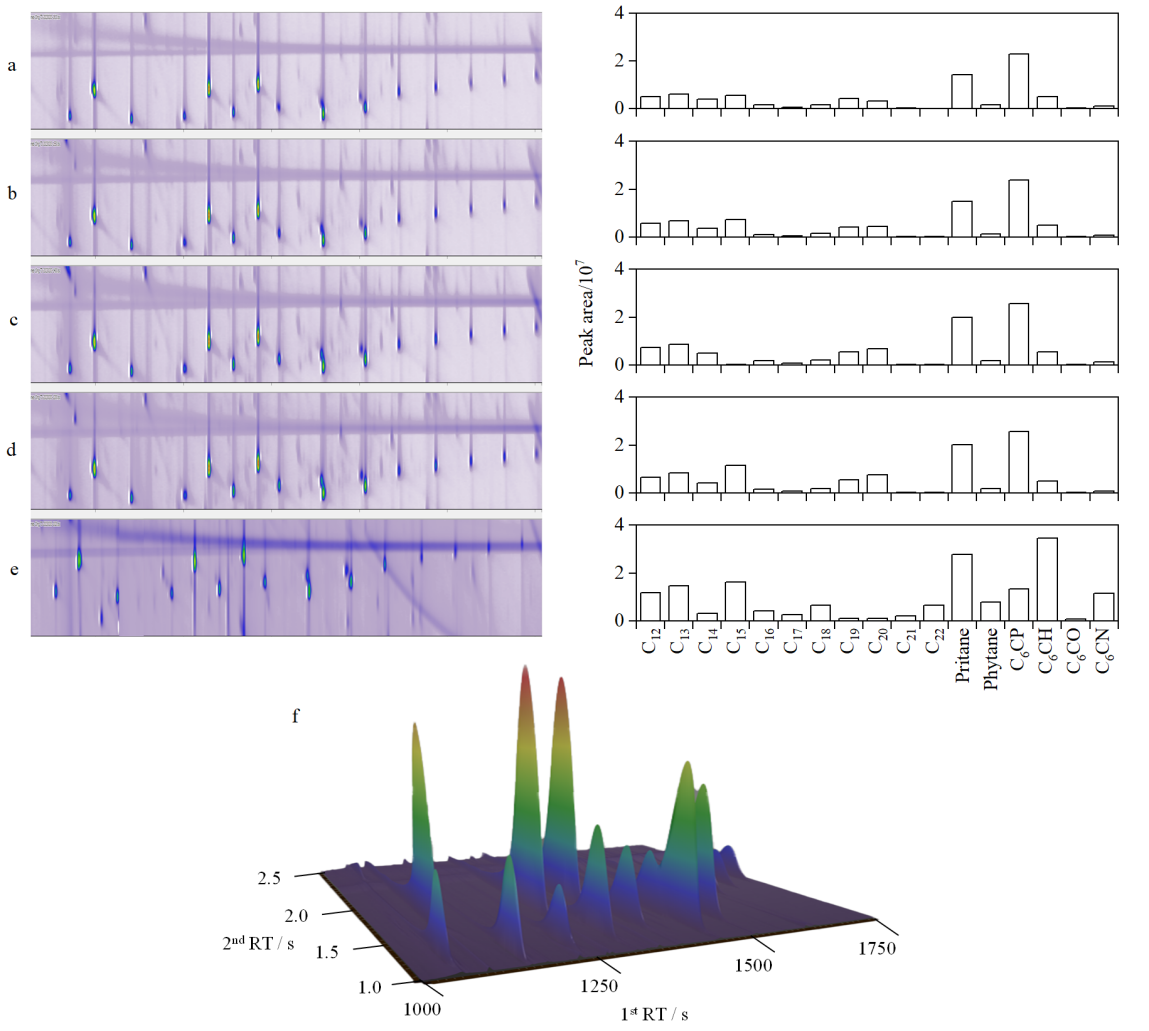
不同吹扫时间下目标物的二维气相色谱图、色谱峰面积及典型样品的三维色谱图

### 2.2 方法验证

#### 2.2.1 线性范围和检出限

采用本方法对69个目标物的系列混合标准溶液进行测定，分别以目标物的添加量为横坐标、目标物与对应内标物的峰面积比值为纵坐标绘制校正曲线。如表4所示，目标物在添加量为1~20 ng/管范围内具有较好的线性关系，相关系数（*r* ^2^）为0.922 3~0.998 4。

**表4:** 69个目标物的回收率、精密度、相关系数和方法检出限

Compound	1 ng （*n*=7）		3 ng （*n*=7）		10 ng （*n*=7）		*r* ^2^	MDL/ （ng/m^3^）
	RE/%	RSD/%	RE/%	RSD/%	RE/%	RSD/%		
C_12_	83.2	25.0	93.0	15.9	82.3	10.0	0.9922	0.0970
C_13_	89.2	32.1	83.1	12.8	84.1	12.8	0.9757	0.1554
C_14_	102.8	21.7	99.3	16.5	108.8	12.8	0.9944	0.1131
C_15_	112.2	20.7	112.0	12.8	117.1	5.0	0.9811	0.2212
C_16_	115.3	28.2	117.8	14.8	119.2	8.2	0.9875	0.2267
C_17_	94.9	21.2	99.9	16.6	96.8	17.3	0.9953	0.1332
C_18_	96.7	17.7	105.1	4.3	100.1	9.9	0.9625	0.2047
C_19_	82.8	21.5	83.7	16.8	87.8	12.8	0.9710	0.0557
C_20_	102.5	31.8	107.7	6.4	82.7	13.8	0.9904	0.0552
C_21_	91.1	24.3	95.1	3.2	87.3	4.5	0.9638	0.1902
C_22_	102.4	27.9	106.3	6.2	105.8	11.5	0.9648	0.1503
Pristane	99.0	23.5	98.7	16.2	101.3	11.2	0.9909	0.3315
Phytane	118.1	16.9	116.1	17.8	121.0	9.2	0.9668	0.1302
C_6_CH	81.0	16.1	72.8	19.4	88.5	14.1	0.9981	0.1621
C_6_CO	119.0	23.7	118.0	11.2	120.0	11.7	0.9984	0.2291
C_6_CN	89.8	14.9	93.1	16.5	104.8	12.2	0.9965	0.1383
1-Dodecene	105.0	15.9	105.8	19.3	112.4	8.9	0.9918	0.0411
1-Tridecene	112.8	29.1	112.1	16.0	116.8	7.1	0.9840	0.1262
1-Cetene	103.3	22.0	103.1	16.6	98.7	10.7	0.9379	0.0566
1-Octadecene	102.4	16.9	111.6	16.4	106.0	11.7	0.9880	0.2222
Nap	113.7	15.2	121.8	17.7	113.1	12.9	0.9752	0.0955
Acp	97.6	23.1	98.2	5.4	101.1	12.9	0.9752	0.1522
Ace	104.5	33.3	103.2	13.8	109.9	3.5	0.9691	0.1448
Flu	108.6	27.9	111.1	7.3	115.5	12.2	0.9821	0.0314
Phe	100.1	13.2	107.2	7.7	107.0	8.0	0.9957	0.0892
Ant	85.9	15.6	89.2	19.1	88.5	9.4	0.9806	0.2564
Fluo	83.1	27.8	87.3	17.0	80.0	8.3	0.9831	0.2188
Pyr	111.8	15.7	110.9	18.3	106.4	5.7	0.9343	0.4101
2-MNap	80.4	4.5	84.2	11.0	92.5	10.4	0.9835	0.0429
1-MNap	102.0	13.5	110.2	15.9	110.5	11.8	0.9872	0.1513
1，3-DMNap	126.2	26.5	123.3	16.8	129.6	7.0	0.9223	0.3185
1，6-DMNap	117.5	26.4	120.1	12.0	117.7	11.9	0.9224	0.3152
1，4-DMNap	129.2	15.8	121.3	16.8	128.7	9.9	0.9316	0.1138
2，3-DMNap	136.0	23.8	119.2	17.1	126.7	12.3	0.9701	0.1133
1，2-DMNap	91.6	23.6	88.0	15.1	88.2	15.2	0.9709	0.1930
1-MT	85.0	33.9	79.5	12.7	87.8	11.9	0.9941	0.3576
1，4-DMT	91.4	29.2	89.3	12.2	93.6	7.3	0.9777	0.2772
2，7-DMT	90.2	26.2	92.9	14.2	99.0	18.6	0.9742	0.3480
2，6-DMT	94.4	24.2	98.6	19.9	91.3	4.5	0.9932	0.1677
1，1，6-TMT	106.5	27.7	106.4	15.2	106.2	7.0	0.9551	0.0600
2‚2‚5‚7-TMT	101.2	30.5	108.7	18.1	98.3	13.1	0.9319	0.2758
1，5，8-TMT	88.6	21.5	84.3	19.8	86.4	5.7	0.9908	0.2092
2，5，8-TMT	87.5	21.5	89.6	17.7	92.1	15.4	0.9908	0.2113
1-N-Nap	82.4	20.9	83.6	10.4	73.6	8.7	0.9730	0.2050
2-N-Nap	84.9	14.3	87.4	18.9	92.7	6.3	0.9955	0.0596
1-PhenylNap	95.0	26.7	99.8	17.1	97.8	6.3	0.9727	0.2759
C_8_-AB	105.1	30.9	109.8	8.0	109.4	11.4	0.9806	0.2074
C_10_-AB	84.7	11.5	83.1	14.0	90.3	14.3	0.9689	0.2497
C_11_-AB	113.8	28.6	121.9	19.5	115.6	11.6	0.9922	0.1176
C_12_-AB	90.5	25.1	88.1	15.3	94.7	12.2	0.9476	0.0497
C_13_-AB	92.6	17.1	92.1	19.4	95.6	7.3	0.9886	0.1563
C_14_-AB	100.9	15.6	101.8	18.0	107.9	15.3	0.9883	0.1482
BTH	123.3	19.9	121.1	19.6	126.3	13.0	0.9541	0.2968
Biphenyl	102.4	23.4	104.1	17.4	107.9	16.2	0.9956	0.3168
HCBD	114.0	25.7	113.0	19.5	119.4	12.8	0.9926	0.1351
1，2，4-TriCB	85.2	25.2	87.5	18.7	90.4	8.7	0.9753	0.1163
1，2，3-TriCB	99.2	14.3	102.8	12.5	98.3	12.4	0.9912	0.0378
2，4，5-TriCB	106.9	14.8	114.8	15.8	113.1	3.5	0.9505	0.0472
1，2，3，4-TetraCB	92.5	23.8	95.7	18.3	94.9	5.4	0.9635	0.1333
1，2，3，5-TetraCB	92.5	23.8	92.1	19.8	99.7	7.2	0.9634	0.1333
1，2，4，5-TetraCB	100.9	11.5	101.3	11.9	94.1	10.4	0.9222	0.0780
PCB	94.8	22.4	97.4	14.7	91.5	5.7	0.9904	0.2329
HCB	94.9	17.7	97.3	14.3	93.9	13.6	0.9976	0.1307
OCS	109.2	19.1	117.0	13.2	122.5	8.0	0.9531	0.0685
DBF	93.6	20.7	101.2	14.8	101.8	13.7	0.9217	0.2151
BP	87.4	14.0	85.8	18.4	88.0	14.9	0.9217	0.2822
DPS	97.5	30.1	97.3	18.2	88.2	13.2	0.9841	0.1239
XT	93.7	21.4	81.8	12.6	101.4	11.2	0.9886	0.0650
2，6-DPP	93.4	13.2	89.7	7.6	97.0	9.1	0.9957	0.0107

向老化后的TD管内添加1 μL 1.2.1节配制的1 mg/L混合标准溶液和1 μL 4.5 mg/L内标混合标准溶液，使目标物的添加量分别为1 ng/管，内标物添加量为4.5 ng/管，进行TD-GC×GC-MS分析。重复7次上述实验，计算其标准偏差，方法检出限（MDLs）为标准偏差与3.14的乘积，以采样体积为2 m^3^计算^［35］^，69个目标物的检出限为0.010 7~0.410 1 ng/m^3^。

#### 2.2.2 加标回收率和精确度

向老化后的TD管内分别添加1 mg/L的混合标准溶液1 μL和4.5 mg/L内标混合标准溶液1 μL，得到目标化合物添加量分别为1、3和10 ng/管、内标物添加量为4.5 ng/管的加标样品，进行TD-GC×GC-MS分析，重复7次。结果表明，1 ng/管添加水平下目标物的加标回收率为80.4%~136.0%；精密度由7次平行分析结果的相对标准偏差（RSD）进行评价^［35］^，1、3和10 ng/管的RSD分别为4.5%~33.9%、3.2%~19.9%、3.5%~18.6%。

### 2.3 实际样品分析

为验证本方法的适用性，利用该方法分析了在上海城区采集的环境大气样品。采样期间IVOCs时间序列变化如图3a所示，总IVOCs质量浓度为8.6~61.1 μg/m^3^（平均值±标准偏差：（22.2±12.8） μg/m^3^），低于本采样点2016年冬季质量浓度（（58.5±27.0） μg/m^3^），但高于2017年夏季质量浓度（（6.8±3.7） μg/m^3^）^［7］^。这一差异可能受采样季节的影响^［36］^，也与采样期间的气象条件、大气氧化性能力、局部源排放等影响有关^［37］^。其中，可识别IVOCs组分的质量浓度为（21.5±12.9） μg/m^3^，占总IVOCs质量浓度的96.2%±4.2%。这一结果与Song等^［38］^基于热调制TD-GC×GC-MS分析餐饮源排放IVOCs可识别组分占比接近（约95%），与Jennerwein等^［39］^基于热调制GC×GC-MS分析的石油中间馏分样品结果相当（95%），略低于Huo等^［40］^采用GC×GC-TOF-MS分析生物质及煤炭燃烧烟气中可识别IVOCs占比（98.5%）。总体来看，本方法与其他热调制技术识别IVOCs组分能力接近，相较于传统一维GC-MS，将可识别IVOCs组分浓度对总IVOCs的贡献从10%~30%提升到90%以上，可识别IVOCs浓度贡献提高了约60%^［7，8，35］^。

**图3:**
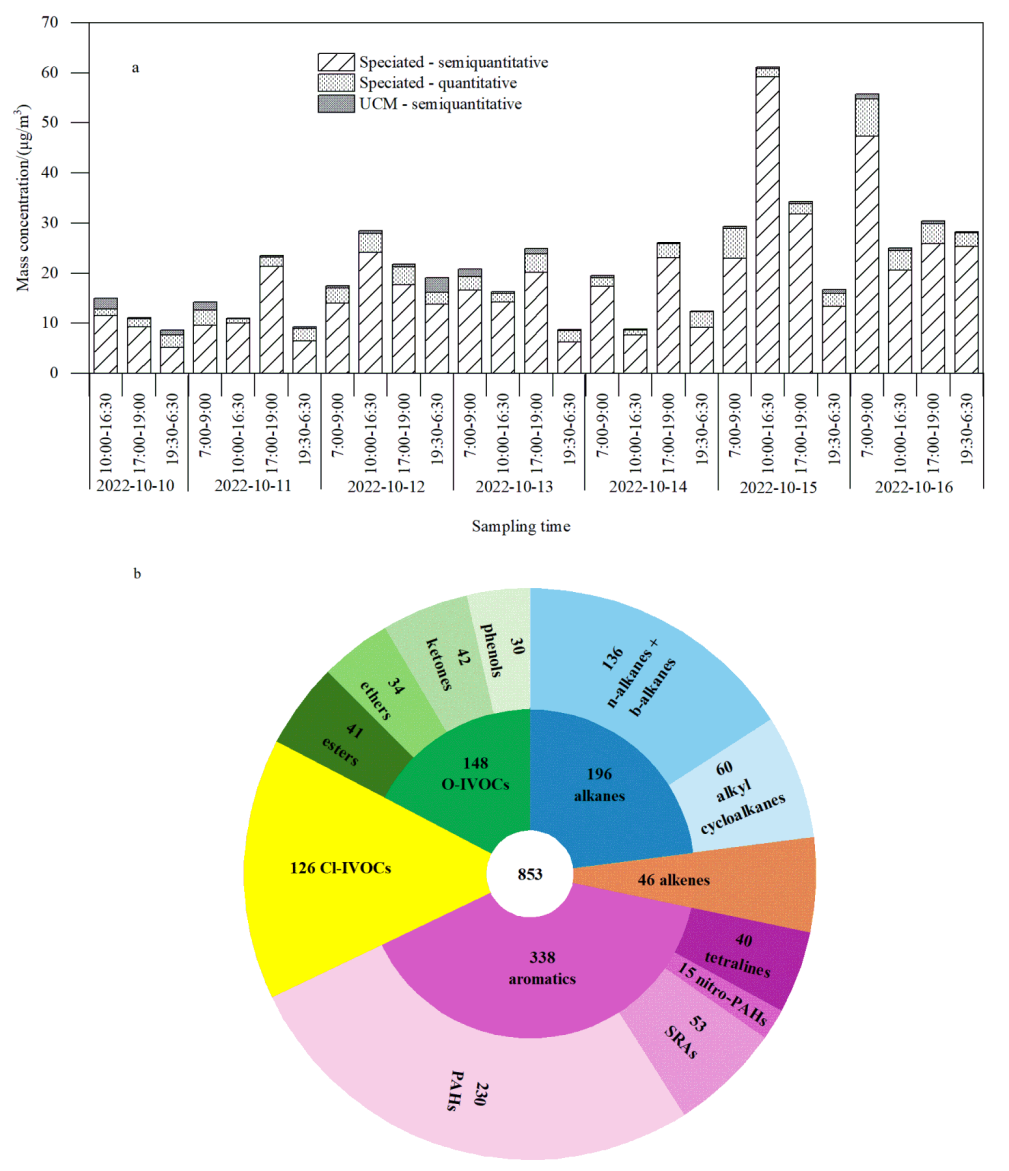
上海城区IVOCs污染特征

采样期间，IVOCs整体上呈早晚双峰排放特征，在本采样地点^［35］^、常州^［41］^、印度德里^［42］^等城市，也观察到类似的现象，这种日变化特征与附近交通影响有关。图3b为本方法识别上海城区环境大气样品中的853个IVOCs组分。根据他们的碳骨架特征分为烷烃、烯烃、芳烃、Cl-IVOCs和O-IVOCs 5类。根据官能团特征不同，识别的IVOCs被分为12个亚类，包括正构与支链烷烃、烷基环烷烃、烯烃、四氢化萘、硝基多环芳烃、单环芳烃、多环芳烃、Cl-IVOCs、酯类、醚类、酮类、酚类。芳烃类有机物是可识别IVOCs的主要组成部分，占可识别组分总数的39.6%。芳烃类有机物由PAHs、SRAs、硝基多环芳烃和四氢化萘4类物质组成，其中识别的PAHs为230个，占芳烃类有机物的68.0%，高于利用一维GC-MS在本采样点识别的17个PAHs^［43］^，以及利用GC-Orbitrap MS识别的中国南海区域有机气溶胶样品中22个PAHs^［44］^，也高于An等^［45］^使用TD-GC×GC-MS识别的北京市区PM_2.5_样品中85个PAHs。识别的烷烃类有机物由正构、支链烷烃和烷基环烷烃组成，共计196个有机物，约占可识别组分的23%，低于Xu等^［28］^使用TD-GC×GC-MS测定的伦敦市区环境空气样品中烷烃（C_13~_C_36_）对总识别有机物的贡献（57%）。其中，识别的正构与支链烷烃IVOCs共计136个，是烷烃的主要组成部分。这一数量远高于加拿大冬季室内样品使用一维GC-MS技术所检测到的烷烃数量（10个）^［46］^，也多于中国南海有机气溶胶中识别的烷烃数量（26个）^［44］^，但与Wu等^［47］^利用GC×GC-MS识别的机动车尾气中的烷烃数量（126~184）相当。识别的148个O-IVOCs组分，包括酯类、醚类、酮类与酚类4类有机物，其中酯类的主要组分为PAEs，共有41个PAEs被识别。Li等^［37］^于2019年利用一维GC-MS检测了本采样点的PAEs污染水平，仅报道了6个PAEs。大气中PAEs的来源主要与建筑材料、消费品的释放相关，这些物质在生产和使用过程中，易通过挥发等过程释放到大气环境中^［37，48］^。本研究还检测识别到126个Cl-IVOCs有机物，也高于An等^［43］^使用一维GC-MS在本采样点识别的7个Cl-IVOCs。根据文献［^7^，^8^，^35^，^43^］，采样期间检测到的芳烃、烷烃和Cl-IVOCs有机物，主要受到周边道路移动源、居民生活排放和上海工业园区排放输送的共同影响。

图4a~e比较了本方法半定量可识别及定量可识别IVOCs总浓度的日变化特征，具体包括烷烃、烯烃、芳烃、Cl-IVOCs和O-IVOCs。结果显示，可识别烷烃（图4a）和烯烃（图4b）的总浓度与定量组分均呈现典型交通源相关的早晚双峰特征，表明这两类化合物的主要来源一致。碳偏好指数（CPI）是用来识别烷烃来源的常用方法之一，通常使用一定碳数范围内奇数碳数正构烷烃的总和与在同一碳数范围内偶数碳数正构烷烃的总和之比进行计算^［21，49］^。CPI值在1~2内表示交通排放突出的城市环境，CPI大于2以上主要受生物源排放影响^［21，50］^。本研究C_12_到C_22_的CPI值为0.43~1.12，进一步表明主要受化石燃料（机动车尾气）排放影响^［33］^。然而，可识别芳烃、Cl-IVOCs和O-IVOCs的总浓度变化趋势与其对应定量组分存在明显差异（图4c~e），这可能由于本方法新识别的这些组分与定量组分具有不同的排放来源^［51-54］^。这一差异进一步表明大气中有机化合物组成复杂、来源广泛，多组分识别有助于准确识别环境大气中有机物的来源。

**图4:**
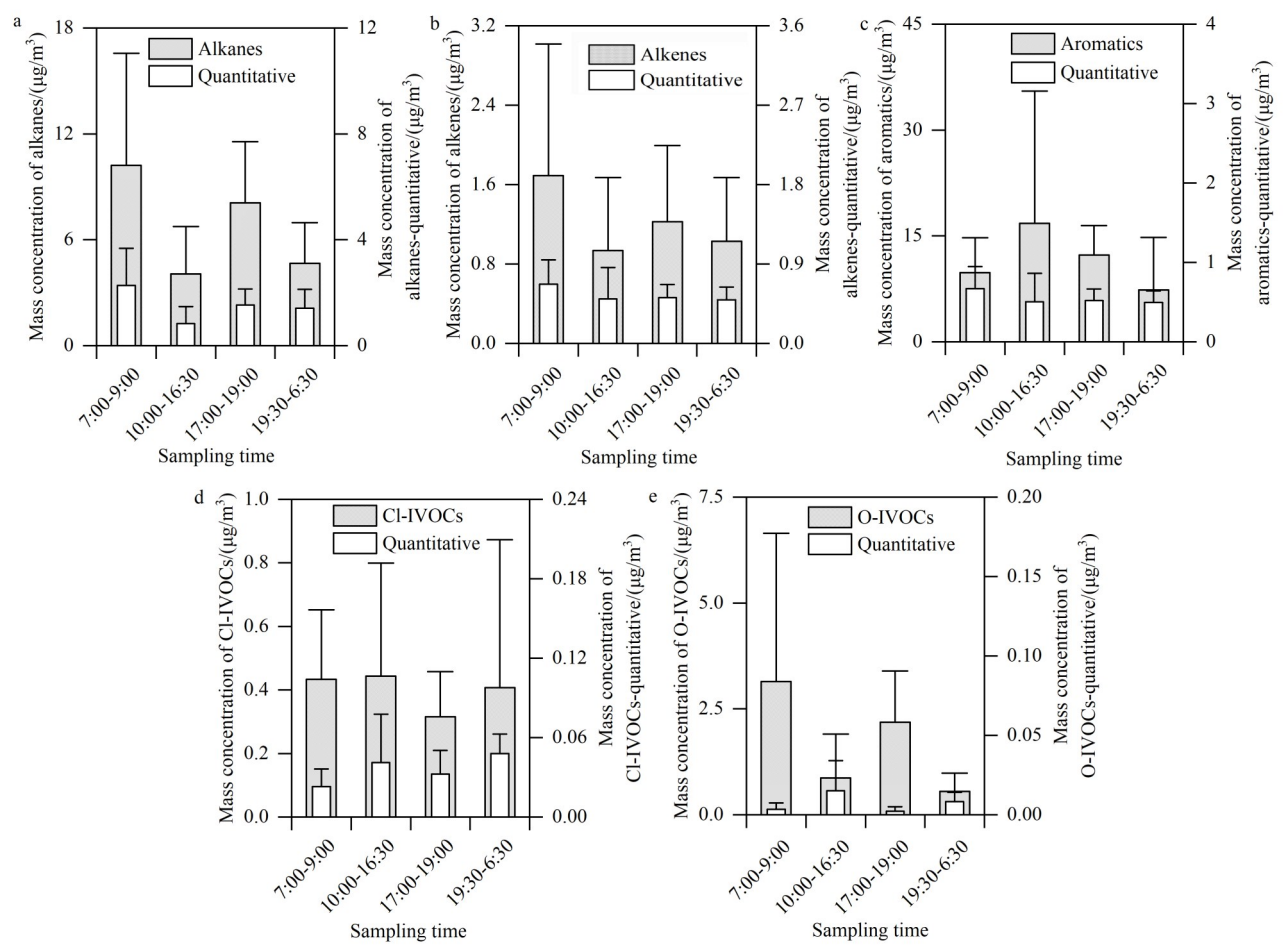
可识别定量组分和可识别组分的比较

## 3 结论

本研究基于TD-气流调制GC×GC-TOF MS技术建立了测定环境大气总IVOCs的分析方法。通过优化气流调制器调制周期的吹扫时间，实现了对IVOCs组分的有效分离和高效识别。该方法具有回收率高、灵敏度高、重复性好等优点。将建立的方法用于测定上海城区环境大气中IVOCs的组成特征。结果显示，上海城区总IVOCs在8.6~61.1 μg/m^3^内变化，共检测到可识别烷烃、烯烃、芳烃、Cl-IVOCs、O-IVOCs等853个组分，占IVOCs总浓度的（96.2%±4.2%）。本研究建立的分析方法对IVOCs组分的识别和定量能力显著提高，可为环境大气复杂IVOCs的准确溯源提供数据支持，并为识别和测定其他介质IVOCs提供技术参考。
